# Brain Molecular Connectivity in Neurodegenerative Conditions

**DOI:** 10.3390/brainsci11040433

**Published:** 2021-03-28

**Authors:** Giulia Carli, Giacomo Tondo, Cecilia Boccalini, Daniela Perani

**Affiliations:** 1 School of Psychology, Vita-Salute San Raffaele University, 20121 Milan, Italy; carli.giulia@hsr.it (G.C.); tondo.giacomo@hsr.it (G.T.); boccalini.cecilia@hsr.it (C.B.); 2 In Vivo Human Molecular and Structural Neuroimaging Unit, Division of Neuroscience, IRCCS San Raffaele Scientific Institute, 20121 Milan, Italy; 3 Nuclear Medicine Unit, San Raffaele Hospital, 20121 Milan, Italy

**Keywords:** [18F]FDG-PET, neurodegenerative diseases, Alzheimer’s disease spectrum, Lewy bodies disease spectrum, brain metabolic connectivity, brain network analysis

## Abstract

Positron emission tomography (PET) allows for the in vivo assessment of early brain functional and molecular changes in neurodegenerative conditions, representing a unique tool in the diagnostic workup. The increased use of multivariate PET imaging analysis approaches has provided the chance to investigate regional molecular processes and long-distance brain circuit functional interactions in the last decade. PET metabolic and neurotransmission connectome can reveal brain region interactions. This review is an overview of concepts and methods for PET molecular and metabolic covariance assessment with evidence in neurodegenerative conditions, including Alzheimer’s disease and Lewy bodies disease spectrum. We highlight the effects of environmental and biological factors on brain network organization. All of the above might contribute to innovative diagnostic tools and potential disease-modifying interventions.

## 1. Introduction

Positron emission tomography (PET) plays a relevant role as a tool able to provide in vivo biomarkers for neurodegenerative diseases, crucial in the diagnosis process [1]. PET measures different molecular processes underlying the pathophysiology of neurodegenerative diseases [2]. These targets include glucose metabolism of cells throughout the well-established radiotracer [18F] 2-fluoro-2-deoxy-D-glucose (FDG), and a broad range of biological and pathological processes, from neurotransmission to amyloid and tau pathology, with corresponding tracers [1].

The progress in the field of neurodegenerative diseases recently brought about a paradigm shift in approaching brain pathology. Thanks to the emergence of robust methods for quantifying the brain’s functional systems [3], the focus of research has shifted from assessing the impact of pathology on local neuron function to investigating the long-distance effect on the interconnected nervous systems [4].

The pathophysiological model of neurodegeneration considers the anatomical and functional relationships between brain regions a relevant subject in neurodegeneration processes [5]. The advent of the “connectivity era” was first characterized by magnetic resonance imaging (MRI)-based brain connectivity analysis due to the wide availability of this tool [4]. Structural connectivity—diffusion tensor images (DTI)—and functional connectivity with functional MRI (fMRI) as well as electroencephalography (EEG) and magnetoencephalography (MEG) allow for the estimation of complex brain networks [6,7]. Recently, a new interest in brain molecular relationships, based on molecular PET data, emerged to define networks throughout radiotracers to detect brain metabolism, neurotransmission, and protein load. Thus, PET-based brain network analysis gradually included brain connectivity measures developed in MRI/EEG/MEG neuroimaging tools [7]. The most diffused molecular and metabolic connectivity methods are the seed-based correlation analysis [8], the independent component analysis (ICA) [9], and methods based on pairwise covariance of brain regions [10,11].

Information on molecular brain network connectivity, as assessed by PET, is still limited, with the few studies mostly focusing on [18F]FDG-PET metabolic connectivity. Here, we review the most recent advances in this emerging field addressing neurodegenerative conditions and the theoretical and methodological framework of brain connectivity. We report on available metabolic and molecular connectivity PET studies in Alzheimer’s disease (AD), alpha-synuclein spectrum, and future directions in the field.

## 2. The Role of PET Imaging in Neurodegenerative Conditions

PET represents a unique tool to detect in vivo several pathophysiological processes, including brain metabolism changes, pathological protein load, neurotransmission integrity, and neuroinflammatory responses [2]. The potential of possible future interventions justifies the massive effort of in vivo molecular research to identify early abnormalities, even years before the clinical onset [12]. PET imaging is today a valuable tool in supporting the diagnosis of neurodegenerative conditions in both clinical and research settings [1]. Moreover, PET imaging may represent a useful tool in screening candidates for clinical trials and may serve as a marker of disease activity in monitoring disease progression [2].

[18F]FDG is the most widely used PET radiotracer, currently employed in clinical and research studies [13]. [18F]FDG-PET signal mirrors neuronal oxidative metabolism and astrocytes glycolysis, mostly reflecting synaptic processes [14]. Several neurodegenerative pathological mechanisms lead to synaptic dysfunction and progressive neuronal loss [2]. Indeed, [18F]FDG-PET hypometabolism reveals alterations in a broad range of neurodegenerative conditions since the very early stage [2]. The main challenge in [18F]FDG-PET analysis concerns the signal quantification methods, which influences diagnostic accuracy [15]. Thus, validated and standardized quantification approaches are needed to provide highly accurate results at the single-subject level, such as brain hypometabolism patterns based on comparisons with a large and well-selected dataset of healthy control [16].

PET also allows abnormal protein deposition measures, representing the pathological hallmark of several neurodegenerative conditions, including AD, frontotemporal lobar degeneration (FTLD), and Lewy bodies disease (LBD) spectrum. Especially in AD, tracer growing availability for detecting underlying pathology has produced a shift to an in vivo biological diagnosis [17].

Since the preclinical and prodromal AD phase, in which symptoms of dementia have not been manifested yet, in vivo detection of amyloid and tau pathology has enabled the identification of candidates for clinical trials [18,19]. Amyloid-PET accurately differentiates AD dementia from FTLD [20] and supports AD diagnosis in individuals with atypical clinical onset [21]. However, it is essential to consider that amyloid-PET reveals brain amyloidosis, which is not invariably associated with dementia [22]. About one-third of healthy elderly individuals have pathological cortical amyloid deposition without showing cognitive impairment [23]. The amyloid-PET positivity classification depends on the selected cut-off, which varies on the basis of the applied quantification method, adding further variability in the outcomes [15]. A weak correlation between cortical amyloid burden and cognitive decline emerged [24], likely because currently available amyloid-PET tracers bind fibrillary insoluble amyloid plaques and not the more toxic amyloid oligomers [25]. Additionally worth noting is that amyloid-PET positivity may also be present in neurodegenerative dementia other than AD, including FTLD and dementia with Lewy bodies (DLB) [26,27]. For all these reasons, using amyloid-PET imaging for screening candidates in clinical trials has been criticized, while the employment of multiple, more specific neurodegeneration biomarkers should be encouraged [1].

Tau-PET imaging, better than amyloid-PET imaging, has the potential to provide staging for AD progression, showing a strict correlation between brain tau protein deposition and measures of atrophy, neurodegeneration, and cognitive decline [28,29]. Moreover, tau pathology correlates with neuronal loss and brain atrophy in tauopathies other than AD [30,31]. However, the selectivity of the currently available tau-PET tracers in non-AD tauopathies still needs further confirmation, and high non-specific binding in subcortical brain structures needs additional care when evaluating tau-PET imaging data [32].

PET imaging allows for the study of brain neurotransmission systems, at both the presynaptic and postsynaptic level, including dopamine, serotonin, noradrenergic, and cholinergic systems [33]. Each neurodegenerative disorder features a prominent disruption in one or multiple specific neurotransmission systems [34]. Specifically, AD patients show a prominent cholinergic depletion; Parkinson’s disease (PD) patients dopaminergic, serotoninergic, and noradrenergic impairments; and DLB patients a severe and widely affected cholinergic and dopaminergic systems [35].

The assessment of the dopaminergic system occurs throughout several radiotracers, such as [18F]Dopa, in order to measure dopamine synthesis; [11C]FE-CIT, for the striatal dopamine transporter (DAT); [11C]raclopride and [18F]Fallypride for binding postsynaptic dopamine D2/D3 receptors [35]. PET molecular imaging also allows quantifying the binding of serotonin receptors (5-HTRs), with the development of successful radiotracers for human studies for 5-HT1AR, 5-HT1BR, 5-HT2AR, 5-HT4R, and 5-HT6R [36]. Regarding the noradrenergic system, PET radioligan’s target is the noradrenaline transporter (NET) [35]. NET is located presynaptically on noradrenergic neurons and noradrenergic projections, where it is responsible for the re-uptake of noradrenaline [35]. In *post-mortem* tissue, cholinergic cell loss detection passes through the choline acetyltransferase activity (ChAT), the enzyme that catalyzes the synthesis of acetylcholine. Although there are no PET radiotracers for ChAT, there are radiotracers for acetylcholinesterase (AChE) or the vesicular acetylcholine transporter—the latter two being able to map acetylcholine cells in the brain with a good correspondence with ChAT [35,37].

## 3. The Viewpoint of Network Dysfunction in Neurodegenerative Diseases

As discussed above, progressive neuronal loss and local changes detected by regional hypometabolism or neurotransmission deficits are well-known hallmarks of neurodegenerative disorders [1,35]. Neurodegeneration affects molecular pathways, local circuits in specific brain regions, and higher-order neural networks [5,38].

A considerable amount of literature on 20th century clinical neuroscience has focused on the localization of psychological processes and clinical symptoms in specific areas of the brain [4]. This approach has provided only a partial analysis of brain function and dysfunction [39]. Other perspectives have emerged during the years. Early in the 20th century, the concept of diaschisis—that local damage to the nervous system can have effects at a distance—has already paved the way to consider the brain as a network [40]. This concept facilitated the development of the “disconnection syndrome theory” by Geschwind [41,42] and the subsequent expansion to a range of clinical symptoms associated with brain connectivity dysfunction [38]. The view that neurological abnormalities may reflect large distance dysfunction rather than local regional changes has a general acceptance because the brain’s pathological perturbations do not involve a single locus. A specific brain area dysfunction will affect structurally or functionally connected regions or through a vulnerable network [4,5,10,43,44]. In this regard, from the 2000s, building on fMRI evidence on resting-state networks, a new theoretical framework was suggested, under the name of “connectomics” [39]. Within the neuroimaging framework, the term “connectivity” is born on behalf of covariations/correlation of a particular signal across brain regions—e.g., blood oxygen level-dependent (BOLD) time courses, PET signal, and EEG signal. The resulting outcomes converge into a common lexicon concerning specific brain covariance profiles, frequently called “brain connectivity”. This framework is also known as the new “brain system biology”, which uses graph theory measures to study brain function and structure characteristics and assumes that the brain must be considered a comprehensive network trait to understand brain functioning [39].

Currently, in the study of neurodegenerative diseases, the concept of the brain as a system of interconnected regions (network) includes two perspectives: one concerns the brain network as a passive target of brain pathology [5], the other as an active participant in the pathology spreading processes [45].

In the “passive” perspective, the damage of high-order brain networks represents the end of a series of related events (a chain reaction), starting from the micro level and ultimately affecting the macroscale level. From a biological point of view, the accumulation of misfolded proteins can cause a cascade of pathological events, including excitotoxicity [46,47], inflammation [48], oxidative stress [49], and other processes [50], which in turn affect the integrity of presynaptic and postsynaptic terminals [5]. The dysfunction of specific brain circuits reverberates to distant brain regions, altering large-scale brain networks [5], resulting in neuroplasticity failure. Chronic changes in synaptic plasticity and neurotransmission can affect activity-dependent signal transduction and gene expression, leading to the neural breakdown and ultimately to neural failure [5,51]. Many factors influence surviving neurons at different stages of the disease. These include the functional status of neurons in the affected area [52], the degree to which alternative neural networks can compensate for the lost neural network [53,54], specific learning strategies to overcome defects [55], and genetic factors such as apolipoprotein E (APOE) [56]. Thus, the pathological process changes an isolated region’s activity and promotes the reorganization of regional interconnection throughout the induction of dedifferentiation with a distributed impact on the brain networks [5].

This framework highlights two different features of brain reconfiguration. The brain is a passive target of the pathology, or it assumes dynamic properties during the network response to neural damage. These assumptions offer an exciting model to assess neuropathological processes underlying neurodegenerative diseases. In parallel, it allows studying the mechanisms by which environmental factors (e.g., cognitive reserve—CR) can modulate neurodegenerative clinical manifestations (e.g., compensation or neural reserve processes).

The hypothesis that neurons and their interconnections play an “active role”—the second perspective—in pathological transmission derives from the observation that a stereotyped pattern of pathological transmission can be detected in every neurodegenerative disease [45]. Subsequent pathology transmission stages identified with the autopsy data indicate that the pathology transmission follows a peculiar topography [57,58]. The autopsy evidence supported by in vivo and in vitro studies has shown that pathological proteins, like prions, are transmitted through synapses together with neuronal interconnections (prion-like) [59]. Animal models show that the protein-spreading pattern depends on the injection site and the neural connector at that specific injection site, not on the type of protein strain [60]. Therefore, the brain network is an active participant in the spread of pathology since it represents the topographical constraint by which the pathology can spread from its initial gathering site [61]. Several neuroimaging studies support this view, showing that pathology and neurodegeneration spread through functional and structural brain networks [62,63]. In this framework, brain connectomics is a useful tool for studying and predicting long-distance pathology’s expansion pattern because pathology expansion strictly depends on the underlying brain connection’s topology [5].

Although reliable evidence supports both the “active” and “passive” concepts of the brain network, there is reason to believe that their relevance may change with the disease’s development. The brain is a highly complex, interconnected network that balances regional isolation and functional specialization through powerful integration [3,4,39]. This balance causes complex and precisely coordinated dynamic changes on multiple temporal and spatial scales [4,43]. The disease transmission mode is extraordinarily complex and related to the highly organized constraints of the underlying neural architecture, the so-called “connectome”. Therefore, network organization fundamentally affects brain diseases, and network science-based connectivity methods are essential for understanding neuropathology. The brain connectivity approach can provide crucial insight into the dynamics of biological changes over time and their interrelationships with the possible clinical progressions in neurodegenerative disorders.

## 4. Network Analysis of Brain PET Imaging

For the past 20 years, the brain network analysis field has had steady scientific production growth [10,11]. Thanks to fMRI studies’ functional connectivity, understanding normal and pathological brain functions has significantly progressed. The first study assessing brain connectivity with data obtained by [18F]FDG-PET dates back to the 1980s [64]. [18F]FDG-PET signal is based on the coupling between synaptic transmission and local glucose consumption, unlike fMRI that detects indirect neural activity, using the amount of oxygen in blood supplying a given brain region. Moreover, the neurovascular coupling—alterations in local perfusion that occur in response to neuronal activity changes—affect fMRI and not [18F]FDG-PET signals. These factors may contribute to the robustness and reproducibility of [18F]FDG-PET connectivity measures. Metabolic connectivity refers to the functional relationships between [18F]FDG-PET measurements in different brain regions. Various analytical approaches exist to examine such relationships: (i) seed correlation or interregional correlation analysis (IRCA), (ii) independent component analysis (ICA), and (iii) regions of interest (ROI)-based approaches (for a more comprehensive review, also see [10,11]).

### 4.1. Seed Correlation or Interregional Correlation Analysis (IRCA)

This voxel-based method relies on the *a priori* selection of ROIs or seeds, extracting the average tracer uptake from that region. Then voxel-wise correlations between average uptake in the seeds and the rest of the brain’s uptake are calculated [8]. Thus, these steps allow for obtaining the connectivity map of the seeds of interest. The seed can be selected in either a data-driven fashion [65,66,67] or on the basis of an *a priori* hypothesis [68,69]. In the data-driven approach, the seeds resulting from previous data analysis are usually the clusters obtained from the first round of univariate analysis [65,66,67]. On the other hand, the seeds’ selection occurs following concrete *a priori* hypotheses [68,69]. The resulting networks have similar topographies to those obtained with resting-state fMRI [70], ensuring a higher discrimination property in some instances.

### 4.2. Independent Component Analysis (ICA)

The ICA is a multivariate approach based on voxel-wise methods, as well as IRCA. Assuming that the PET signal figured as a mixture of statistically independent components, ICA has its foundation in PET signals’ multivariate decomposition across the brain [70]. This method allows for identifying coherent brain networks (for example, the resting state networks) in a data-driven manner without the need to select a specific seed/ROI in advance. However, the number of components to be extracted need to be set by the investigator. The selection of those components with pathophysiological or anatomo-functional meaning is crucial, discarding pure statistical noise components. ICA represents the method of choice for connectivity analysis using fMRI data. Some studies investigated ICA’s applicability on [18F]FDG-PET data for large-scale network estimation [70,71,72]. Although the main resting-state networks are identifiable in both two imaging modalities—fMRI and [18F]FDG-PET data—there is a lack of a complete spatial overlap [71,72]. This mismatch suggests that fMRI and [18F]FDG-PET may capture different aspects of network integrity.

### 4.3. Regions of Interest (ROI)-Based Approaches

ROI-based approaches allow for computing a “connectivity matrix” starting from selecting a set of target regions. ROIs can emerge according to *a priori* hypothesis, i.e., ROIs belonging to a specific anatomo-functional system of interest or a data-driven approach, i.e., ROIs covering the whole brain. Partial correlation analysis and sparse inverse covariance estimation (SICE) are two widely used ROI-based approaches [11]. The former allows for estimating the degree of linear association between each couple of selected ROIs, factoring the contribution of all remaining ROIs. Partial correlation analysis overcomes the limitations of simple correlation analysis, which captures paired information and cannot characterize the effect of multiple interacting brain regions [73]. SICE finds the estimated value of inverse covariance, thereby indirectly providing a measure of partial correlation [4,73]. The advantage of SICE is that it can estimate molecular connectivity even if the number of subjects included in the analysis is less than the number of ROIs (which is relatively frequent in PET studies) [73]. This aspect is essential for connectome assessment because connectivity studies select many ROIs covering the entire brain. Once the SICE algorithm estimates the whole brain connection matrix, it is possible to calculate the graph theory indexes, e.g., the brain hubs and modules, and changes in node and global network characteristics [44].

## 5. Molecular and Metabolic Connectivity in Neurodegenerative Conditions

### 5.1. Alzheimer’s Disease Spectrum

AD represents the most common cause of neurodegenerative dementia, accounting for an estimated 60%–80% of cases [74]. The intracellular oligomers and the extracellular accumulation of the protein amyloid-beta (Aβ) and the intra-neuron deposition of abnormal tau protein are the disease’s pathological hallmarks [17]. These pathological events lead to progressive neuronal dysfunction and neurodegeneration, which clinically manifest as progressive cognitive decline [17]. Since AD pathological changes seem to start decades before symptoms arise, much of the current research focuses on detecting brain AD-related changes when clinical symptoms are subtle or not yet manifested, using in vivo biomarkers. For these reasons, a framework for a biological definition of AD emerged, identifying three broad phases: the preclinical AD, mild cognitive impairment (MCI) due to AD, and AD dementia, on the basis of the detection of biomarkers Aβ, tau pathology, and neurodegeneration [17].

The assumption that brain regions whose metabolism is correlated are functionally interconnected [64] has its first application in AD patients, who showed the loss of bilateral connection between the entorhinal cortex and several cortical regions compared with normal controls [75]. Brain metabolic connectivity application underlines brain network alterations in demented patients and in non-demented cognitively impaired subjects to detect early and specific signatures of neurodegeneration in the whole AD clinical spectrum and aged cognitive unimpaired subjects [68,76,77,78,79].

**Figure 1:**
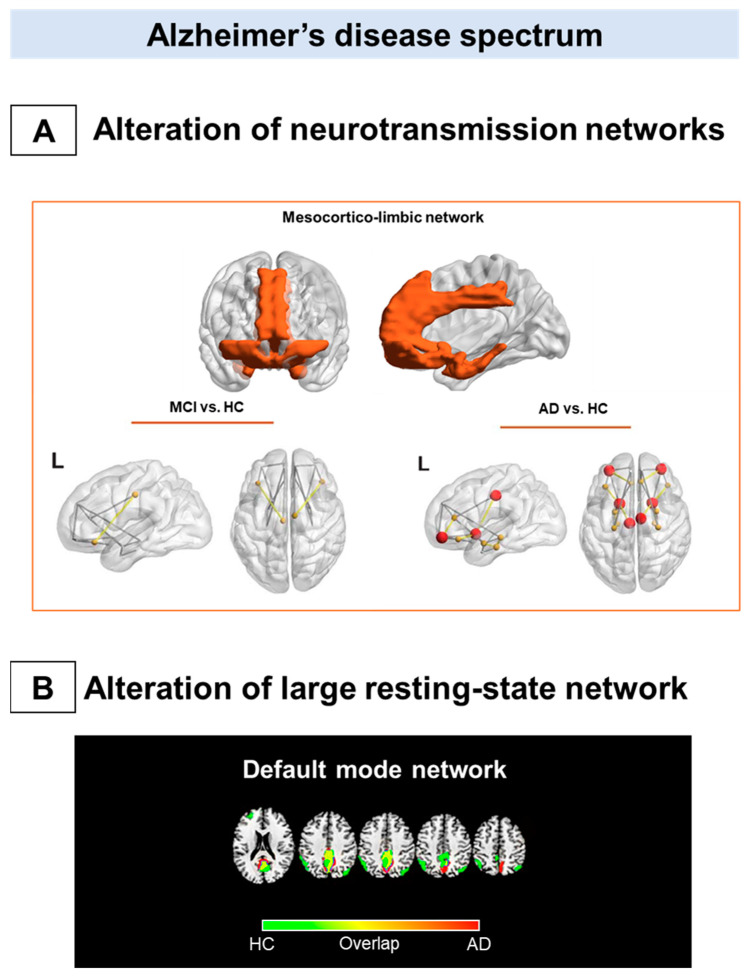
Metabolic connectivity alterations in Alzheimer’s disease spectrum. The figure summarizes the main metabolic connectivity findings concerning Alzheimer’s disease clinical spectrum. The neurotransmission systems (**A**) and the resting-state networks (**B**) characterized by different levels of metabolic connectivity impairment in Alzheimer’s disease (AD) and mild cognitive impairment (MCI). (**A**) There is a paucity of studies assessing neurotransmission alteration throughout the metabolic connectivity approach. Recent metabolic connectivity evidence demonstrates the mesocorticolimbic dopaminergic system’s involvement in AD, which is stage-dependent [90]. The mesocorticolimbic dopaminergic network involves the ventral tegmental area, the ventral striatum, the medial-temporal cortex, and the medial prefrontal cortex (top panel). Compared with healthy controls, AD patients show a disruption in the connections between the ventral striatum and medial frontal and temporal cortices (bottom right panel). These alterations become less evident, moving from the dementia phase to the prodromal phase along the AD continuum (bottom left panel). (**B**) The default mode network disruption is consistently reported as the prominent metabolic connectivity signature of AD. In healthy controls, the DMN comprises the posterior cingulate cortex, the precuneus, the angular gyrus, and the medial prefrontal cortex (green map). A disruption involving the posterior cingulate cortex/precuneus and frontal connections occurred in AD (red map). Panel A modified from Journal of Alzheimer’s Disease, Vol number 78, L. Iaccarino, A. Sala, S.P. Caminiti, L. Presotto, D. Perani, In vivo MRI structural and PET metabolic connectivity study of dopamine pathways in Alzheimer’s disease, Pages No 1–14., Copyright (2021), with permission from IOS Press 2021. Abbreviations: MCI = mild cognitive impairment, AD = Alzheimer’s disease; vs. = versus; L = left; HC: healthy controls, DMN: default mode network.

PET metabolic connectivity is closely associated with data derived from fMRI studies [80] and, in AD, has primarily focused on the investigation of resting-state networks. The default mode network (DMN) is one of the most studied networks in both functional and metabolic connectivity studies, and this is not surprising, given that it includes the AD signature regions [1]. The posterior cingulate cortex’s involvement and the precuneus, crucial hubs of the DMN, have been repeatedly reported in several studies investigating metabolic connectivity in AD [68,77,78]. There is a general agreement in considering the alteration of DMN, a specific metabolic signature of the AD clinical spectrum (Figure 1B). An increasing gradient of DMN damage is present along the AD continuum, starting from the MCI condition to the mild dementia stage [79,81,82]. The breakdown of metabolic connections between the posterior cingulate cortex and hippocampus seems to be a common feature in different AD subtypes but most pronounced in the amnestic type [78].

On the other hand, atypical AD presentations may show more distinct features. Atypical variants are quite frequent in the early-onset AD (EOAD), which affects people younger than 65 years, who show greater disease severity and faster disease progression than the typical late-onset AD (LOAD) [83]. When compared with controls, LOAD and EOAD have presented different metabolic connectivity features, wherein the former showed altered connectivity involving temporo-occipital regions and the latter showing cingulate gyri and occipital areas [84]. EOAD patients also showed more extensive global network disruptions, correlating with the severity of dementia as quantified by the clinical dementia rating scale [84]. By investigating the association between neuropsychiatric symptoms and brain metabolic connectivity dysfunctions in a large sample of EOAD patients, Ballarini and colleagues showed behavioral abnormalities in EOAD associated with specific dysfunctional changes in brain metabolic connectivity, suggesting both a disruption of the DMN and increased connectivity of the anterior salience network as compared to controls [68]. The largest disarranging of brain networks observed in EOAD might be due to the more extensive pathology alterations. These include high deposition of amyloid and tau proteins and severe neuroinflammatory responses, as revealed in a recent study investigating the relationship between microglial activation and alterations of brain network connectivity in EOAD [67].

Multivariate approaches are particularly appropriate when exploring the effect of risk factors for AD, including age and genetic susceptibility. APOE-ε4 genotype has been associated with changes in metabolic connectivity already in healthy controls, confirming its role as a risk factor for AD [85]. Older adults had changes in metabolic brain networking compared to younger subjects, and the alterations were more evident in subjects with APOE-ε4 genotype and brain amyloidosis, with a degree similar to that shown in AD [85]. In AD patients, genotype seems to affect the functioning of the DMN, specifically with a distinctive pattern of reduced metabolic connectivity in the ventral DMN, correlating with episodic memory scores [86]. Aβ pathology, which represents the AD spectrum’s pathological hallmark, is strongly associated with abnormal patterns of metabolic connectivity involving the temporal–parietal regions [87]. By modelling cortical Aβ as a continuous variable in a sample including healthy controls, MCI patients, and AD patients, Carbonell and colleagues showed that the cumulative effect of Aβ deposition was related to reduced metabolic connectivity in AD signature regions [87]. These findings confirm that Aβ pathology plays a role in disrupting metabolic interactions between regions.

Metabolic connectivity can also differentiate neurodegenerative dementias. Using SICE, Titov and colleagues revealed different and characteristic patterns of altered metabolic connectivity in AD and FTLD patients, with an overall accuracy of 83% [88]. Specifically, AD and FTLD patients showed, when compared with controls, pathological connections in the parietal lobe and the frontal and temporal lobes, respectively [88]. When comparing AD and FTLD patients, higher altered connections between the parietal and the temporal lobe were found in the first group [88]. In a very recent study, Imai and colleagues analyzed the metabolic connectivity in AD and DLB patients using the graph-theoretical method [89]. Decreased connectivity resulted in both groups compared with healthy controls, but DLB patients showed more limited and more profound network disruption than AD, with the posterior cingulate and the Heschl’s gyri representing the most affected regions [89]. These data underline the importance of multivariate approaches in the differential diagnosis of dementing disorders.

Of note, one study has investigated in vivo neurotransmission systems using the metabolic connectivity approach in AD [90]. In this study, alterations in morphology and network topology, specifically of the dopaminergic mesocorticolimbic pathway, were demonstrated, crucially considering the reported involvement of brain dopaminergic neurotransmission associated with neuropsychiatric and cognitive symptoms in AD [90]. No alterations of the mesocorticolimbic connectivity were found in the MCI group, suggesting that molecular connectivity changes appear in a more advance disease stage (Figure 1A).

Molecular connectivity studies focusing on AD pathology are still sparse and have tried to delineate amyloid and tau spreading, depending on the dynamic brain network interactions. Sepulcre and colleagues identified hub regions, including the medial temporal lobes and the hippocampus, whose amyloid pathology was associated with further amyloid deposition in cortical regions, such as orbitofrontal and temporal–parietal cortices. The described spreading mechanism was evident both in AD patients and in cognitively normal subjects [91].

### 5.2. Lewy Bodies Diseases Spectrum

PD is the most common form of Parkinsonian syndromes [92]. Its clinical diagnosis occurs with the presence of significant motor symptoms, including rigidity, akinesia/bradykinesia, postural instability, and rest tremor [92]. Neuropathologically, PD is characterized by the presence of Lewy bodies (LB) and Lewy neurites (LN) containing alpha-synuclein oligomers [93]. Other neurodegenerative disorders (i.e., DLB and multiple system atrophy (MSA)) [93] share similar neuropathological hallmarks, and together with PD are called alpha-synucleinopathies. Moreover, isolated rapid eye movement (REM) sleep behavior disorder (iRBD) represents the prodromal stage of full-blown alpha-synucleinopathies [94]. There are several PET metabolic connectivity studies in PD and DLB [66,95,96,97,98,99,100,101], and less evidence in iRBD [95,102] and MSA [103,104].

**Figure 2:**
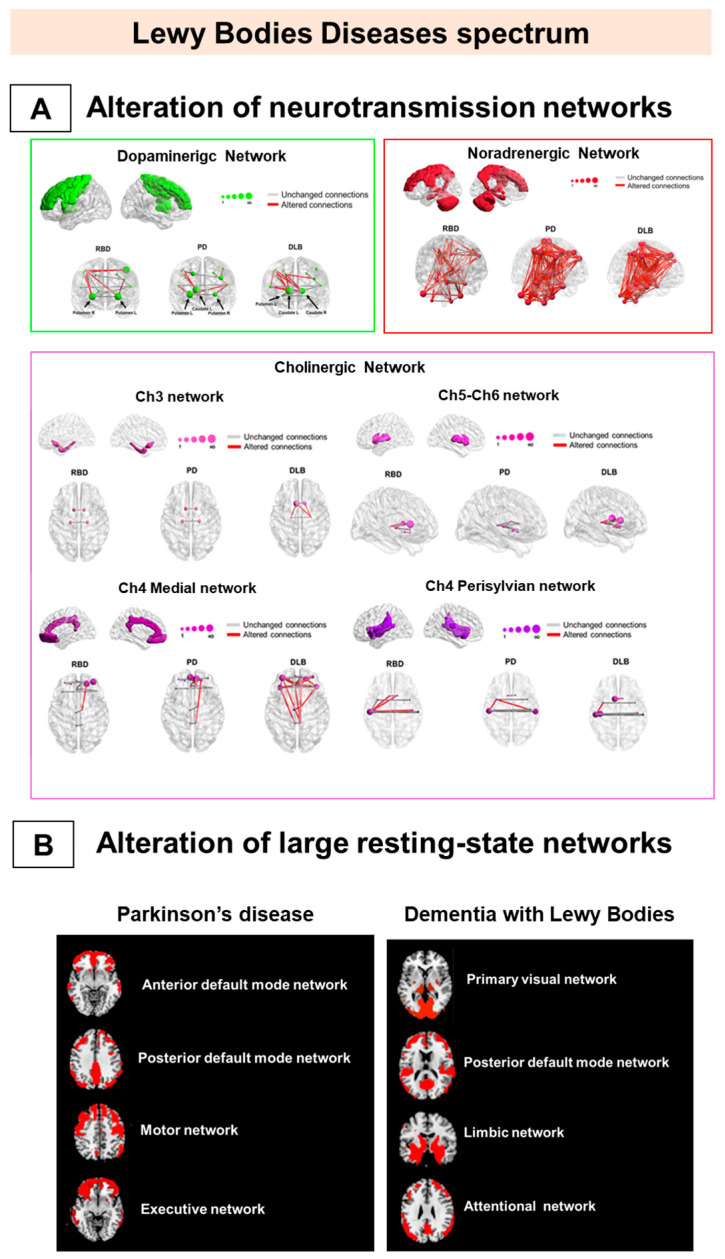
Metabolic connectivity alterations in Lewy bodies disease spectrum. The figure represents the main metabolic connectivity findings in Parkinson’s disease (PD), dementia with Lewy bodies (DLB), and isolated rapid eye movement (REM) sleep behavior disorders (iRBD). The figure depicts the neurotransmission networks (**A**) and the large-scale resting-state network alterations (**B**). (**A**) DLB and PD share connectivity changes, mainly in noradrenergic and cholinergic (Ch)4 perisylvian (P) cholinergic networks. The iRBD and DLB groups show high similarity in noradrenergic and Ch5-Ch6 cholinergic networks. IRBD and PD show a high degree of similarity in the noradrenergic network. Finally, the dopaminergic network impairment is limited and localized in iRBD and moderate-to-severe in DLB and PD. (**B**) PD is characterized by alteration of the frontal component of anterior default mode network DMN, posterior DMN, and motor and executive networks (right), and DLB by alteration of the posterior component of PVN, pDMN, and limbic and attentional networks (left). All the evidence supports that alpha-synucleinopathies should be considered multisystem disorders since the prodromal stage. Panel A modified by Carli et al. (2020) [95] with the permission of Elsevier 2021. Abbreviations: RBD = REM sleep behavior disorder, PD = Parkinson’s disease; DLB = dementia with Lewy bodies; DMN: default mode network; Ch5-Ch6 = cholinergic Ch5-Ch6 divisions networks; Ch4 medial = cholinergic medial Ch4 division network; Ch4 perisylvian = cholinergic lateral perisylvian Ch4 division networks; Ch3 = cholinergic Ch3 division network; Ch1-Ch2 = cholinergic Ch1-Ch2 division network.

As mentioned above, disease-specific brain network alterations, mainly involving the posterior DMN, characterize AD already in the prodromal phase. Conversely, in PD and DLB, connectivity alterations of resting-state large-scale networks revealed a widespread derangement of the brain connectome, suggesting them as multi-network neurodegenerative disorders (Figure 2B) [96,97,98].

PD patients are characterized by a connectivity derangement in several resting-state systems, specifically in the frontal components [97]. These networks involve the attentional network, the anterior DMN, and the executive and motor networks [97]. These networks are strictly related to the critical pathological events leading to neurodegeneration (e.g., dopaminergic depletion) and the consequent clinical manifestations during the disease course. For example, the metabolic connectivity in the DMN is preserved in early-stage PD patients, showing, however, a progressive disruption with the emergence of mild cognitive impairment occurring in PD later stages [100]. DMN alteration seems to be closely associated with PD dopaminergic depletion [100] and partial restoration of DMN connectivity after dopaminergic therapy [100].

Moreover, for the executive network, prefrontal and orbitofrontal networking impairment might follow the frontal–striatal dopaminergic imbalance [105]. Although the role of frontal lobe dysfunction in executive deficits is recognized in PD, it is not clear whether it contributes to cognitive decline and dementia development [106]. The early predictor of PD dementia, instead, is the posterior cortical damage [107]. The dual syndrome hypothesis suggests that prefrontal and striatal dysfunctions are features of stable non-dementia PD patients and that the posterior cortex and temporal lobe derangements are signs of rapid cognitive decline [107]. The pathological involvement of the frontal networks in PD seems to be associated with the neuropsychiatric manifestation, typically characterizing this condition [108]. A recent study tried to map common PD non-motor symptoms—i.e., impulsivity and hypomania—using seed-based metabolic connectivity analyses [108]. Impulsivity and metabolism showed an association in the medial part of the right superior frontal gyrus, brain structure functionally connected with brain regions belonging to the anterior DMN. The impulse control disorder in PD patients was associated with severe impairment of the mesocorticolimbic metabolic connectivity [99] and a functional loss of covariance, as measured with DaTSCAN SPECT imaging, between basal ganglia and frontal associative cortex [109]. This evidence further highlights the link between dopaminergic deficits and network integrity in PD.

In DLB, metabolic connectivity evidence showed a severe involvement of the posterior cortical, the limbic, and the attention networks, consistently with the pathological and clinical heterogeneity intrinsic to this condition [98]. DLB connectivity alterations in large-scale resting-state networks are strongly related to the clinical symptoms [98]. Visual hallucinations (VH) are associated with impaired connectivity in posterior DMN, the attention network, and visual networks [66,98]. The biological explanation for these findings points to cholinergic damage, leading to the breakdown of the functional relationship among the calcarine cortex, lateral occipital cortex, and parietal cortex [110]. These studies demonstrated that the neural correlates of complex neurological symptoms, such as VH, encompass multiple large-scale brain networks. Visual attention and executive, visuoperceptive, and visuospatial deficits in DLB patients are associated with specific large-scale network connectivity changes [98]. Visual attention and visuospatial neuropsychological deficits are related to impaired metabolic connectivity in the primary and associative visual networks, whereas executive dysfunction is related to a frontal metabolic connectivity impairment [98]. Of note, the association between visual selective attention and integrity of metabolic connectivity in the primary visual network crucially supports the hypothesis that visual cortex desynchronization is a key factor in DLB visual attention deficits.

The multisystem derangement of large-scale networks in PD and DLB is also confirmed by metabolic connectivity studies targeting neurotransmission systems [95,96,97]. Alpha-synuclein aggregations play a crucial role in the neurotransmitter impairment observed in these syndromes, affecting different systems—the dopaminergic, noradrenergic, and cholinergic sytems [111]. Degeneration of dopaminergic nigro-striatal neurons is the pathological hallmark of PD and DLB associated with the typical motor impairment [93]. According to neuropathological findings, several pieces of metabolic and molecular connectivity data have consistently reported relevant connectivity reconfigurations within the nigro-striato-cortical dopaminergic network in PD and DLB [95,96,97]. Moreover, metabolic connectivity alterations are present in the noradrenergic network in PD and DLB, in agreement with *post-mortem* observations [112]. Of note, limited metabolic cholinergic network alterations characterize PD, while a widespread and severe metabolic connectivity impairment features DLB [95].

These data led to a comparative picture of the metabolic connectome in the alpha-synucleinopathies (Figure 2). PD and DLB condition both revealed consistencies and substantial differences. PD patients show an extensive decrease in connectivity in frontal regions and a compensatory connectivity increase in occipital and posterior cortical regions [97]. In contrast, an occipital connectivity impairment is the hallmark of DLB, together with a more preserved frontal connectivity [97]. The different patterns of metabolic connectivity alterations might reflect vulnerabilities of the cholinergic system in PD and DLB [105], leading to disease-specific patterns of dysfunction [113,114], as confirmed by recent metabolic connectivity evidence [95]. In this regard, PD and DLB patients present common and disease-specific neurotransmission network reconfigurations. Both clinical syndromes show moderate-to-severe alterations in the nigrostriatal dopaminergic [95,96,97] and noradrenergic networks [95], whereas the DLB condition presented more severely affected cholinergic networks [95] (Figure 2A). PET molecular connectivity in DLB was also investigated at different stages, as measured by DaTSCAN SPECT [115]. The connectivity analysis showed alterations in the limbic circuits and basal ganglia with differences in DLB with mild or intermediate/severe dopamine deficiency. Connectivity alterations increased slightly in DLB with mild dopamine deficiency but degenerated strongly when dopamine deficiency became intermediate or severe [115]. All the above findings indicate that PD and DLB conditions must be considered complex and multisystem disorders.

Metabolic connectivity findings indicate that the whole alpha-synuclein spectrum is a multisystem neurotransmission disease since the early stages (Figure 2A). Notably, in iRBD, today considered the early stage of DLB, patients showed severe impairment of noradrenergic network connectivity, as well as an initial derangement of the cholinergic networks [95]. IRBD shared some cholinergic alterations with DLB, indicating an early occurrence and a role in this system’s phenotypic expressions. Conversely, the nigro-striato-cortical dopaminergic network featured limited connectivity changes in iRBD. This metabolic signature is consistent with molecular neuroimaging findings, demonstrating a nigrostriatal dopamine innervation within normal limits in iRBD cases [116]. These connectivity data also represent an in vivo confirmation of Braak staging of alpha-synuclein propagation [58]. Two other in vivo studies support the Braak staging [58] hypothesis in PD and DLB conditions only, reporting the brain regions’ metabolic disconnection affected by alpha-synuclein spreading [96,97].

Despite a common molecular pathological substrate and a backbone of shared neuronal vulnerabilities, alpha-synucleinopathies show significant clinical differences, particularly regarding the timing and severity of symptoms. Since connectivity is a marker of the clinical phenotype, distinct network dysfunction patterns—related to the severity and extent of neurodegenerative processes [44]—might help explain these differences.

## 6. Biological and Environmental Factors Influencing Neurodegenerative Connectivity Changes

The clinical phenotypes in neurodegenerative condition are dependent on some fixed (e.g., sex, genotype) and flexible (e.g., gender, education, occupation, and leisure activity) factors. This assumption is related to the concept of brain reserve (BR), whereby education, occupation, other lifestyle factors, and inherited factors may contribute to differences in brain structure and function that modulate resistance against the neurodegenerative processes [117]. The cognitive reserve (CR) hypothesis states that the underlying pathology is more severe in higher reserve individuals [118]. Several studies, employing [18F]FDG-PET univariate approaches, have supported the CR hypothesis by reporting a significant association between high reserve proxies and severe hypometabolism in different neurodegenerative conditions [119,120,121,122,123,124]. The brain’s capacity to cope with neurodegeneration passes through the brain network architecture’s neural plasticity properties [5]. The brain connectivity approach, thereby, can provide the opportunity to capture momentous events linked to the neural networks’ complex dynamics and can best capture experience-based plasticity, revealing how BR copes with brain dysfunctions. Changes in brain network organization parallels the neural responses to damage, with critical maladaptive processes (e.g., transneuronal degeneration and dedifferentiation) and resources (e.g., compensation) that enable adaptation [5].

From a methodological standpoint, interconnected brain regions can be characterized by an increase or a decrease in metabolic connectivity, reflecting different brain functioning. Specifically, the decrease of metabolic connectivity indicates a functional disconnection between regions, while metabolic connectivity increase indicates a functional coupling between regions [10,11,43]. However, increases in neural activity may represent compensatory and pathogenic mechanisms underlying pathological processes [5]. Indeed, caution is needed when interpreting connectivity increases also to understand CR neural implementation.

Evidence has supported the CR modulation on metabolic connectivity in different neurodegenerative diseases, such as DLB and AD. Metabolic connectivity alterations are present in several large-scale networks in AD and DLB, in association to reserve proxies such as education [65,120,123], occupation [120], and bilingualism [124]. For example, increases in metabolic network connectivity result in relevant resting-state networks, namely, the DMN, the executive networks, and the language networks, in association with education and occupation in AD patients, with significant gender differences such as prominent frontal executive recruitment neural resources in females [123]. Bilingualism is an essential reserve source in AD, wherein bilingual patients have more preserved metabolic connectivity in the DMN and executive networks than monolinguals and show compensation in the frontal less affected circuits related to the amount of second language usage [124]. The robust functional connectivity related to bilingualism condition in AD indicated compensation in the anterior frontal network. These metabolic findings in AD are consistent with the hypothesis that connectivity increases in brain regions that are still unaffected and functionally active may underlie compensatory processes similar to functional connections seen in healthy models.

Recent evidence suggests that education and occupation can modulate resting-state network metabolic connectivity in DLB [120]. Education seems to act through compensatory mechanisms since it showed a specific influence on the executive, attentive, and posterior DMN in which a highly educated DLB sub-group engaged the anterior brain regions to cope with more severe brain posterior pathology. On the other hand, occupation acts through neural reserve mechanisms, with a specific effect on anterior DMN and associative visual network, in subjects with high occupational attainment. Thus, CR induces brain plasticity, resulting in neural reserve, making the brain more resistant to pathological changes and preserving the cognitive functions. Moreover, the same article investigated the occupation considering specific occupational profiles—different metabolic connectivity patterns emerged in association with occupational proxies’ and types [120].

These studies underline how cognitive reserve proxies can cope with the diseases thanks to neural reserve mechanisms and the recruitment of compensatory neural networks.

Other factors influence the metabolic connectivity changes and help to explain the heterogeneous clinical manifestation of neurodegenerative disorders. Sex differences in brain structure and function through sex-determining genes and hormonal factors have important implications for brain-based disease risk [125]. A variety of broader socio-demographic factors associated with gender differences, including role expectations and social attitudes, influence the risk, course, and clinical expression of neurodegenerative diseases [125]. Emerging studies have reported gender effects on the structural and functional connectivity in health and disease, suggesting a significant gender influence on the patterns of neuronal networking, possibly underlying cognitive and behavioral gender differences [126]. Some studies applied the metabolic connectivity approach to explore such gender differences [123,127,128,129]. In AD patients, DMN, executive and language networks’ brain metabolic architecture revealed gender differences. Females showed a more extended frontal–parietal connectivity in all these networks than males, who had more pronounced differences in the executive and language networks [123]. In AD, the interaction between gender and the body mass index (BMI) was recently assessed to study its modulation on resting-state networks connectivity. The anterior DMN and salience networks showed reduced connectivity in AD females with high BMI, but not in males, leading to the conclusion that high BMI may negatively affect AD females’ brains [128]. All the above information suggests a multi-factorial modulation of brain connectivity in AD.

In PD, a recent study assessed gender differences in the molecular architecture of dopaminergic systems linked to specific clinical features, using partial correlations analyses. A broader alteration of metabolic connectivity within the nigro-striato-cortical network was the hallmark in male patients, whereas a deeper reconfiguration of the mesolimbic system characterized the female PD sample [129]. These findings fitted the estrogen-induced neuroprotection hypothesis and the different gender-related clinical manifestations observed in PD [130].

Brain metabolic connectivity analyses could reveal how several factors influence and modulate the neurodegenerative processes. Further studies are needed to investigate the link between gender-specific connectivity patterns and clinical manifestation to explore some brain disorders’ gender-specific nature. This more comprehensive approach may impact treatments and the targeting of modifiable risk factors.

## 7. The Elusive Side of Brain Connectivity Approach: Limits and Challenges

Brain connectivity approaches have enabled the scientific community to improve knowledge about neurodegenerative diseases and related pathological processes. However, many definitional and methodological issues are still present, highlighting an elusive side of connectivity conceptualization.

Several studies over the years have claimed that they were examining something akin to functional connectivity with multiple types of data, such as EEG measures, fMRI time series, and [18F]FDG-PET data [131]. However, the various types of data used to evaluate functional interactivity differ in many aspects, including spatial and temporal resolution and the ability to directly measure neuronal activity and neurobiological aspects of the brain. For example, the synchronous fluctuations in low-frequency BOLD signal between different brain regions (measures of functional connectivity) provide an indirect measure of neural activity by using the amount of oxygen in blood supplying a given brain region. It follows that brain connectivity measures cannot be necessarily backed with specific biological processes. For that reason, opinions on the use of the term “functional connectivity” are somewhat divided [131]. To a certain point, referring to complex brain networks or covariance patterns—terms derived by the domain of a branch of discrete mathematics, known as graph theory—any reference to strictly biological processes could be avoided, thus escaping the use of “functional connectivity” terminology.

The [18F]FDG-PET signal (based on the coupling between synaptic transmission and local glucose consumption) may represent a step forward in this regard. However, PET-based brain network estimation presents some issues as well. One restriction consists of the limited spatial resolution of PET, which make the small brain nuclei challenging to study, e.g., raphe nuclei, locus coeruleus, substantia nigra, and ventrotegmental area. Indeed, it is always necessary to verify that the volume of the area included in the analysis, especially in ROI-based methods, is not less than three times the full width at half maximum (FWHM) of the scanner’s spatial resolution. This spatial resolution “cut-off” is considered the lower limit value to avoid confusing effects such as blurring or spill-over [132,133].

Brain networks result from multivariate analysis methods to estimate metabolic covariance for [18F]FDG-PET and molecular covariance for other PET data in different brain regions [64,131,134]. The actual computational approaches used to assess these parameters differ between investigators [10,11,135]. Thus, there is a strong need for validation studies to demonstrate the reproducibility of results obtained with these methods. For example, a recent study has shown good reproducibility of complex brain networks measured with different radiotracers, including [18F]FDG, obtained with an ROI-based correlative approach and graph theory, suggesting general applicability within typical experimental settings [135]. More studies employing other analytical methods are needed to consolidate and extend these findings. The ultimate goal should be to standardize and harmonize the computational methods to make different studies comparable.

Finally, due to the inherent “static” nature of PET images, the vast majority of brain network PET-based results are based on the group-level analysis. Indeed, PET images do not possess the temporal component that characterizes, instead, fMRI data [10]. This limitation makes impossible a within-subject “fMRI-like” analysis of PET images. Thus, further crucial research is needed in order to quantify PET molecular and metabolic covariance changes at a single subject level, a top priority in the field of neurodegenerative diseases (see [11]).

## 8. Future Directions

The increasing application of molecular connectivity techniques has allowed for remarkable advances in understanding neurodegenerative diseases’ dysfunctional mechanisms. Molecular approaches based on PET imaging provide evidence upon associations of brain metabolism and neurotransmitter systems. These techniques may help further investigate the complex network architecture in response to pharmacological and rehabilitative treatments. Customized networks result from functional metabolic data from PD patients who received gene therapy, and new treatment-induced brain circuits have been identified [136]. This innovative application of molecular and metabolic covariance patterns paves the way to track underlying disease progression and treatment effects at the systems level, providing insight into underlying biological mechanisms. Another primary objective for future work is to establish a reliable application for connectivity analyses in clinical settings, such as methodological developments for individual assessment and data replication. In this way, complex brain networks signatures might help clinical diagnose and choice of therapy. In this view, the combination of various imaging modalities represents an appealing approach to provide a comprehensive picture.
